# Assessment of Cytotoxic and Genotoxic Responses to an Ipfencarbazone-Based Herbicide in Human Peripheral Lymphocytes *İn Vitro*

**DOI:** 10.3390/cimb48060565

**Published:** 2026-05-28

**Authors:** Ahmet Ali Berber, Cansu Akbulut, Esra Yıldız, Sinem Öztürk, Şefika Nur Demir, Nurcan Berber

**Affiliations:** 1 Vocational School of Health Services, Çanakkale Onsekiz Mart University, 17100 Canakkale, Türkiye; nberber@comu.edu.tr; 2 Department of Biology, Faculty of Science, Sakarya University, 54050 Serdivan, Türkiye; cansua@sakarya.edu.tr (C.A.); ebilgin@sakarya.edu.tr (E.Y.); 3 Department of Biology, School of Graduate Studies, Çanakkale Onsekiz Mart University, 17100 Canakkale, Türkiye; sinemozturk@stu.comu.edu.tr; 4 Department of Molecular Biology and Genetics, Faculty of Science, Ataturk University, 25240 Erzurum, Türkiye; sefikademir@atauni.edu.tr

**Keywords:** ipfencarbazone, ipfencarbazone-based herbicide (IPF-BH), genotoxicity *in vitro*, cytotoxicity-associated genotoxic-like effects, human lymphocytes, mitotic index, micronucleus assay, comet assay

## Abstract

This study evaluates the cytotoxic and genotoxic-like potential of an ipfencarbazone-based herbicide formulation (IPF-BH; commercial product Hokuto, containing 250 g/L of the triazolinone herbicide ipfencarbazone) in human peripheral lymphocytes *in vitro* across a concentration range of 62.5–1000 µg/mL. Cytotoxicity was monitored via the mitotic index (MI), while cytogenetic damage was assessed using the cytokinesis-block micronucleus (MN) assay and the alkaline comet assays. Comparisons were performed using one-way ANOVA, followed by Dunnett’s post hoc test, against the negative control. Results indicated a concentration-dependent cytotoxic effect, with a marked reduction in MI observed at all tested concentrations (*p* < 0.001). MN frequency was significantly elevated at concentrations ≥125 µg/mL, whereas the 62.5 µg/mL concentration did not induce significant micronuclei formation. The comet assay revealed increased DNA damage parameters (tail length, tail intensity (%), and tail moment) across the tested concentration range, albeit with a non-monotonic profile for tail length and tail intensity. These findings suggest that IPF-BH exposure is associated with marked cytotoxicity and a genotoxic response in this *in vitro* model at concentrations within the OECD 487-acceptable cytotoxicity window, together with cytotoxicity-associated genotoxic-like effects at strongly cytotoxic concentrations in human peripheral lymphocytes under *in vitro* conditions. Because IPF-BH is a commercial formulation, and no direct mechanistic endpoints (e.g., reactive oxygen species, mitochondrial transmembrane potential, lipid peroxidation, glutathione) were measured, and because the present design was performed without exogenous metabolic activation (no S9 supplementation), the observed effects cannot be unambiguously attributed to ipfencarbazone alone or to a defined mechanism of action; extrapolation to *in vivo* genotoxicity requires complementary +S9 and rodent *in vivo* follow-up studies.

## 1. Introduction

Pesticides are extensively employed in modern agriculture to enhance crop productivity by controlling a wide spectrum of pests, including weeds, insects, fungi, and rodents [1]. Among these, herbicides play a pivotal role in suppressing weed growth and improving crop yield and quality [2]. However, their widespread and often indiscriminate use has raised significant concerns regarding environmental contamination and human health risks. Exposure to pesticides can occur directly during application or indirectly through contaminated food, water, and environmental matrices [3]. Epidemiological and experimental studies have associated chronic pesticide exposure with various adverse health outcomes, including cancer, metabolic disorders, respiratory diseases, and neurodevelopmental impairments [4]. Moreover, individuals occupationally exposed to pesticides frequently exhibit cytogenetic damage, highlighting the genotoxic potential of these compounds [5]. One of the principal mechanisms underlying pesticide-induced toxicity involves the generation of reactive oxygen species (ROS), which disrupt cellular redox balance, impair enzymatic systems, and induce oxidative DNA damage, including single- and double-strand breaks [6]. Consequently, comprehensive evaluation of the mutagenic, genotoxic, cytotoxic, and carcinogenic properties of pesticides is essential for accurate risk assessment and regulatory decision making.

Triazolinone herbicides represent an important class of compounds widely used for effective weed management. These herbicides are generally known to act as protoporphyrinogen oxidase (PPO) inhibitors, leading to the accumulation of phototoxic intermediates such as protoporphyrin IX, which ultimately results in membrane disruption and cell death [7]. Several members of this class, including sulfentrazone, have been reported to induce DNA damage and genomic instability in both plant and mammalian systems, demonstrating their potential genotoxic and cytotoxic effects [8,9]. These findings underscore the importance of evaluating the toxicological profiles of newly developed herbicides within this chemical class.

Ipfencarbazone is a relatively novel triazolinone herbicide developed for use in paddy rice cultivation, particularly for controlling problematic weeds such as *Echinochloa* spp. [10]. Unlike many triazolinone herbicides, ipfencarbazone exerts its herbicidal activity through the inhibition of very-long-chain fatty acid (VLCFA) elongase, thereby disrupting lipid biosynthesis, impairing cell division, and ultimately leading to plant death [11,12]. Its high efficacy and selectivity toward rice crops have contributed to its increasing agricultural application [13]. However, despite its agronomic advantages, regulatory and toxicological data present a complex, and at times, contradictory safety profile. According to safety data sheets and regulatory assessments, ipfencarbazone is classified as a Category 1B carcinogen and a specific target-organ toxicant following repeated exposure, with reported effects on the liver, hematopoietic system, and urinary bladder. An acceptable daily intake (ADI) of 0.00099 mg/kg body weight/day has been established, reflecting concerns regarding long-term exposure [14,15].

In contrast, earlier toxicological evaluations conducted during its development reported low acute toxicity in rodents, minimal irritation or sensitization effects, and no evidence of mutagenicity, teratogenicity, or carcinogenicity in standard *in vivo* studies [10]. This apparent discrepancy between regulatory classifications and experimental findings highlights the need for further investigation into the compound’s biological effects. Additionally, environmental monitoring studies have detected ipfencarbazone residues in aquatic organisms and food products, such as fish (0.01 mg/kg) and agricultural commodities, indicating potential for bioaccumulation and indirect human exposure [16,17]. Its relatively slow degradation under anaerobic conditions further suggests persistence in paddy field environments, increasing the likelihood of long-term ecological and human health impacts [18].

Genotoxicity assessment of pesticides is inherently complex, as results often vary significantly depending on the experimental model and methodology employed. Large-scale evaluations of agrochemicals have demonstrated that compounds frequently yield negative results in bacterial reverse mutation (Ames) assays, while producing positive responses in mammalian cell-based assays, such as the mouse lymphoma assay (MLA) or the HPRT test [19,20]. For example, certain compounds tested at concentrations up to 100 µg/mL have shown positive mutagenic responses *in vitro*, yet failed to induce genotoxic effects in *in vivo* rodent models, including comet and transgenic mutation assays [21,22,23]. These inconsistencies are often attributed to differences in cellular sensitivity, particularly in the use of rodent-derived cell lines with compromised p53 function, which may overestimate genotoxic risk compared to that of primary human cells [24,25]. Such variability complicates the extrapolation of *in vitro* findings to human health risk and emphasizes the importance of using biologically relevant systems.

For ipfencarbazone, the available in silico toxicological data further contribute to this uncertainty. Computational models, including Toxtree, Vega-QSAR, and T.E.S.T., have yielded conflicting predictions, with some classifying the compound as toxic and others suggesting potential mutagenicity, while carcinogenicity predictions remain inconsistent [26]. These findings contrast with regulatory reports indicating no classification for germ cell mutagenicity based on available experimental data [27]. Such discrepancies highlight the limitations of predictive models and reinforce the necessity of experimental validation.

Although standard genotoxicity testing strategies typically integrate assays such as the Ames test, micronucleus assay, and mammalian cell gene mutation tests, no single method is sufficient to capture the full spectrum of DNA damage [28,29]. Furthermore, dose-dependent effects of ipfencarbazone have been observed in environmental and agricultural contexts, such as residue accumulation in rice reaching 14.71 mg/kg following application at 312.50 g active ingredient per hectare [30]. Despite these observations, detailed mechanistic studies investigating oxidative stress induction, DNA strand breaks, or chromosomal damage in human cells remain scarce.

Genotoxicity refers to the ability of chemical agents to damage genetic material, leading to mutations, chromosomal aberrations, and genomic instability, which may ultimately result in carcinogenesis or heritable genetic defects. Reliable assessment of genotoxic potential, therefore, requires the use of complementary endpoints targeting different levels of genetic damage. Widely accepted methods include the mitotic index (MI) for evaluating cytotoxicity and cell proliferation, the micronucleus (MN) assay for detecting chromosomal damage and mitotic disturbances, and the comet assay for assessing primary DNA strand breaks at the single-cell level.

Despite increasing agricultural use, regulatory concern, environmental persistence, and conflicting in silico and experimental data, there is a notable lack of studies directly evaluating the genotoxic and cytotoxic effects of commercial ipfencarbazone-based herbicide formulations in human cells. In particular, data derived from primary human peripheral lymphocytes, considered a highly relevant model for human health risk assessment, are absent from the current literature. This critical knowledge gap underscores the need for comprehensive *in vitro* investigations to clarify the genetic and cellular risks associated with IPF-BH exposure. Therefore, the present study aimed to evaluate the cytotoxic and cytogenetic effects of an ipfencarbazone-based herbicide (IPF-BH) in human peripheral lymphocytes using the comet assay, the micronucleus test, and mitotic index analysis, thereby providing essential data to support more accurate toxicological risk assessments and safer agricultural practices.

## 2. Materials and Methods

### 2.1. Test Substance: Ipfencarbazone-Based Herbicide (IPF-BH)

The test material used throughout this study was the commercial ipfencarbazone-based herbicide formulation Hokuto (HOKKO CHEMICAL INDUSTRY Co., Ltd., Tokyo, Japan), containing 250 g/L of ipfencarbazone as the declared active ingredient, together with co-formulants whose identities and concentrations were not disclosed by the manufacturer. To clarify that the study was performed using a commercial formulation rather than an analytical-grade reference standard, the test substance is hereafter consistently referred to as the ipfencarbazone-based herbicide (IPF-BH). All concentrations reported in this manuscript (62.5, 125, 250, 500, and 1000 µg/mL) refer to the IPF-BH formulation as applied to the cultures. The possible contribution of co-formulants to the observed effects is addressed in the Section 4.3.

### 2.2. Collection of Blood Samples

Peripheral venous blood was collected from four healthy donors (two males and two females), aged between 20 and 25 years who were non-smokers and non-alcohol users, for all cytogenetic assessments. These individuals had no history of chromosomal fragility, recent viral infections, exposure to known mutagenic substances, or drug treatments in the past two years and had not undergone ionizing radiation in the past six months. This study was approved by the Non-Interventional Clinical Research Ethics Committee of Çanakkale Onsekiz Mart University (Approval No: 2024-190).

### 2.3. Mitotic Index Test

The LD_50_ dose of the active ingredient ipfencarbazone is reported to be 2000 mg/kg in rats [10]. Based on this data, the application concentrations of IPF-BH were initially determined as 125, 250, 500, 1000, and 2000 µg/mL. The selection of the highest test concentration was guided by the reported acute toxicity profile of ipfencarbazone and by the cytotoxicity-based criteria recommended in OECD Test Guidelines 487 (*in vitro* mammalian cell micronucleus test) and 489 (*in vivo* mammalian alkaline comet assay), which indicate that the highest concentration should ideally produce no more than approximately 55 ± 5% cytotoxicity, while concentrations leading to complete or near-complete cytotoxicity should be excluded from genotoxicity evaluation. In the preliminary mitotic index study, no dividing cells were observed at 2000 µg/mL, indicating complete cytotoxicity at this level. Accordingly, this concentration was excluded from subsequent cytogenetic analyses, and the working concentration range was rescaled to 62.5, 125, 250, 500, and 1000 µg/mL of IPF-BH. The retained concentrations were considered appropriate for the present study because they encompassed a clear cytotoxicity–response trend while preserving an interpretable number of dividing and binucleate cells, thereby allowing concentration–response analysis of cytogenetic endpoints. The interpretation of effects observed at the highest retained concentrations is, however, treated with caution and is discussed under the Section 4.3.

A heparinized whole blood sample (0.2 mL) was added to 2.5 mL of Chromosome Medium B (Biochrom AG, Berlin, Germany, Cat No: F 5023) and incubated at 37 °C for 72 h. Cultures were treated with IPF-BH at concentrations of 1000, 500, 250, 125, and 62.5 µg/mL, along with a negative control and a positive control (Mitomycin-C; MMC, 0.2 μg/mL, PanReac AppliChem, Darmstadt, Germany, Cas No: 50-07-7). Colchicine (0.06 μg/mL) was added 2 h before harvesting, and cells were exposed to the test substance for a treatment period of 24 h (from the 48th to the 72nd hour of culture) for mitotic index assessment.

At the end of the culture period, tubes were centrifuged at 1200 rpm for 10 min, and the supernatant was discarded. A hypotonic solution (5 mL, 0.075 M KCl) was added, followed by incubation at 37 °C for 30 min. After another centrifugation, the supernatant was discarded. Each tube was then treated with 5 mL of cold fixation solution (3:1 methanol:acetic acid) and incubated at +4 °C for 45 min, with the process repeated three times. After the final centrifugation, cell pellets were homogenized and spread onto microscope slides. The slides were stained with 5% Giemsa stain (Merck, Darmstadt, Germany, HS No: 3204 19 00) (pH 6.8) for 15–20 min, then mounted with Entellan to prepare permanent slides for examination.

### 2.4. Micronucleus Assay

The blood culture was initiated for 72 h, as in the mitotic index test. At the 24th hour, the test substance (IPF-BH), negative control (sterile distilled water), and positive control (Mitomycin C; MMC, PanReac AppliChem, Cas No: 50-07-7) were added, giving an effective IPF-BH exposure of approximately 48 h (from the 24th to the 72nd hour of culture), in line with the cytokinesis-block micronucleus assay protocol recommended in OECD Test Guideline 487 for peripheral blood lymphocytes treated without exogenous metabolic activation. At the 44th hour, 5.2 µg/mL Cytochalasin-B (PanReac AppliChem, Cas No: 14930-96-2) was added as a cytokinesis inhibitor. At the end of the culture period, the tubes were centrifuged at 1000 rpm for 10 min, the supernatant was discarded, and 5 mL of cold 0.075 M KCl was added, followed by incubation at +4 °C for 5 min. After another centrifugation, 5 mL of cold fixation solution was added, the mixture was incubated at +4 °C for 15 min, and then centrifuged again. The fixation process was repeated, and then a fixative containing 1% formaldehyde was added before the final centrifugation. After removing the supernatant, the remaining cells were homogenized with a pipette and spread onto microscope slides. The cell culture was dropped onto slides pre-cleaned with 1 N nitric acid and stored in 70% ethanol at −20 °C. The slides were stained with 5% Giemsa solution for 13–15 min and mounted with Entellan to create permanent preparations for examination.

### 2.5. Alkaline Comet Assay in Isolated Human Lymphocytes (İn Vitro)

Lymphocytes were isolated using Biocoll separation solution. Cell viability was confirmed to be over 99% using the trypan blue exclusion test. A total of 100 μL of blood was added to 900 μL of PBS in Eppendorf tubes, suspended, and incubated on ice for 10 min. Then, 100 μL of Lymphoprep was added to the bottom of the tubes and centrifuged at 1060 rpm, +4 °C for 3 min. The isolated lymphocytes were distributed in 100 μL aliquots, and IPF-BH, along with the control groups (sterile H_2_O: negative control; 100 µM H_2_O_2_: positive control), were added, followed by a 1-h incubation at 37 °C, which represents the short-term exposure protocol routinely applied for the alkaline comet assay in isolated peripheral lymphocytes. After incubation, the tubes were centrifuged at 3000 rpm for 5 min, the supernatant was discarded, and the cells were resuspended in 100 μL of PBS. A total of 100 μL of lymphocyte culture was mixed with 75 μL of Low Melting Agarose (0.65% in PBS, kept at 37 °C), spread onto agarose-precoated slides, and covered with 24 × 60 mm coverslips. The slides were incubated at +4 °C for 20–25 min to allow agarose solidification.

Following solidification, the coverslips were carefully removed, and the slides were immersed in freshly prepared, ice-cold lysis solution consisting of 2.5 M NaCl, 100 mM Na_2_EDTA, and 10 mM Tris-HCl, with 1% (*v*/*v*) Triton X-100 and 10% (*v*/*v*) DMSO added immediately before use; the pH of the final lysis solution was adjusted to 10.0 with concentrated NaOH. Lysis was performed for 1 h at +4 °C in the dark.

After lysis, the slides were drained, rinsed gently, and placed horizontally in a chilled electrophoresis tank filled with freshly prepared alkaline electrophoresis buffer (300 mM NaOH and 1 mM Na_2_EDTA; pH > 13). An alkaline unwinding step was performed for 20 min at +4 °C in the dark, allowing the DNA to unwind and alkali-labile sites to be expressed. Electrophoresis was then carried out for 20 min at 1 V/cm (approximately 25 V, ~300 mA, adjusted by buffer volume) at +4 °C in the dark.

After electrophoresis, the slides were neutralized by gentle washing in 0.4 M Tris-HCl buffer (pH 7.5) at +4 °C for 3 × 5 min, then drained and stained with ethidium bromide (20 µg/mL, 50 µL per slide) under a coverslip and incubated at +4 °C for 15–20 min in the dark. Finally, the prepared slides were examined under a fluorescence microscope at 40× magnification. For each concentration, 100 cells per donor and a total of 400 cells per concentration group (across the four donors) were analyzed. “Hedgehog”/“ghost” comets (cells with very small or absent heads and a diffuse halo, generally considered indicative of irreparable damage or apoptotic/necrotic cell death) were excluded from quantification, in line with the consensus recommendations for the *in vitro* alkaline comet assay [31,32]. Comet images were analyzed using CometScore software (v1.5, TriTek Corp., Sumerduck, VA, USA), and DNA damage was quantified using tail length, tail intensity (%), and tail moment parameters.

The selected assays (mitotic index, cytokinesis-block micronucleus assay, and alkaline comet assay) were chosen because they are internationally widely used methods for the comprehensive assessment of cytotoxicity, chromosomal damage, and DNA strand breaks in human lymphocytes.

### 2.6. Slide Evaluation

In determining the mitotic index (MI), a total of 4000 cells were examined for each concentration, with 1000 cells per donor (four donors). The mitotic index was determined by calculating the ratio of dividing cells to the total number of cells, expressed as a percentage. Relative MI (%MI of treated group/%MI of negative control × 100) was also computed at each concentration as a relative cytotoxicity index, in order to align our interpretation with the OECD 487 cytotoxicity threshold of approximately 55 ± 5%. Pre-exposure cell viability was independently confirmed at the lymphocyte isolation step using the trypan-blue exclusion test (>99% viability, as stated in Section 2.5). The combined use of MI, relative MI, the cytostasis parameter NDI (defined below), and pre-exposure trypan-blue viability provides a multi-parameter cytotoxicity readout, although we acknowledge that, in line with OECD 487 recommendations, MI alone is not a complete cytotoxicity indicator (the limitations are detailed in Section 4.3, and complementary post-treatment cytotoxicity assays are recommended in Section 4.4 for future studies). For determining the micronucleus (MN) frequency, 500 binucleate cells were scored per donor for each concentration, corresponding to a total of 2000 binucleate cells per concentration group across the four donors. We acknowledge that the per-donor scoring number used here is below the 2000 binucleate cells/donor recommended by OECD Test Guideline 487; this aspect is addressed in the Section 4.3.

Cytostasis was evaluated using the nuclear division index (NDI), which is a commonly applied cytostasis parameter in cytokinesis-block micronucleus assays, particularly with human peripheral lymphocytes. For NDI determination, 1000 cells were counted per donor for each concentration to score the proportion of cells containing one, two, three, or four nuclei. The nuclear division index was calculated as follows:

NDI = [(1 × N_1_) + (2 × N_2_) + 3 × (N_3_ + N_4_)]/N (1)

where N_1_–N_4_ represent the number of cells with one to four nuclei, respectively, and N is the total number of cells scored. It should be noted that the current OECD Test Guideline 487 recommends the cytokinesis-block proliferation index (CBPI) as the preferred cytostasis parameter for *in vitro* micronucleus assays. The absence of CBPI calculation in the present study is acknowledged as a methodological limitation and is discussed in the Section 4.3. In line with OECD 487, MN frequencies were interpreted jointly with NDI and with the corresponding relative MI value at each concentration, so that MN responses occurring under conditions of excessive cytotoxicity could be flagged as potentially cytotoxicity-driven rather than as evidence of direct genotoxicity.

### 2.7. Statistical Evaluation

All statistical analyses were performed using IBM SPSS Statistics (Version 22, IBM Corp., Armonk, NY, USA).

The normality of the residuals was assessed using the Shapiro–Wilk test, and homogeneity of variances across groups was assessed using Levene’s test. For each cytogenetic endpoint—mitotic index (MI), micronucleus (MN) frequency, nuclear division index (NDI), and the comet assay parameters of tail length, tail intensity, and tail moment—differences across groups were evaluated using one-way analysis of variance (ANOVA). Where the overall ANOVA F-test was significant (*p* < 0.05), pairwise comparisons of each IPF-BH treatment group against the common negative control were performed using Dunnett’s post hoc test, which is specifically designed for many-to-one comparisons against a shared control and controls the family-wise Type I error rate.

In addition, the global concentration–response relationship between IPF-BH concentration and each measured parameter was characterized by linear regression analysis, with Pearson correlation coefficients reported for descriptive purposes. Statistical significance is reported in all tables using the standardized notation * *p* < 0.05, ** *p* < 0.01, and *** *p* < 0.001 (Dunnett-adjusted versus negative control), with additional in-table annotations indicated by lower-case superscript letters (a, b, c), where necessary.

## 3. Results

In this study, the *in vitro* cytotoxic and cytogenetic effects of IPF-BH on human lymphocytes were evaluated using the mitotic index (MI), the cytokinesis-block micronucleus (MN) assay, and the alkaline single-cell gel electrophoresis (comet) assay. The data obtained from the 24-h applications of IPF-BH in the mitotic index test on human lymphocytes are presented in Table 1. Compared with the negative control, the mitotic index decreased at all tested concentrations following 24 h of treatment, and these reductions were statistically significant (Dunnett post hoc test, *p* < 0.001). Relative MI values (treated/negative control × 100%) decreased from 78.6% at 125 µg/mL to 50.4% at 1000 µg/mL, indicating that the higher IPF-BH concentrations exceeded the OECD 487 cytotoxicity threshold of approximately 55 ± 5%.

**Figure 1:**
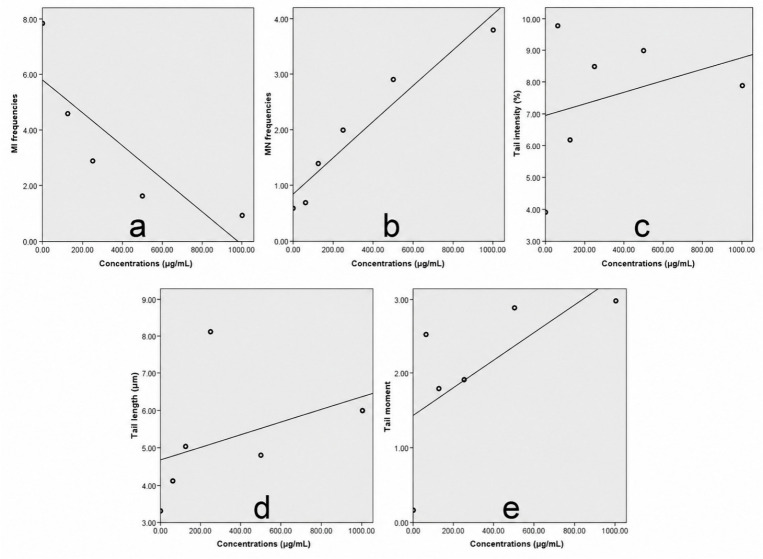
Dose-dependent variation in MI frequencies (**a**): in 24-h r = −0.84; (**b**): dose-dependent change in MN frequencies r = 0.96; dose-dependent change in comet test parameters (**c**): tail intensity r = 0.32; (**d**): tail length r = 0.35; (**e**) tail moment r = 0.67.

Across the IPF-BH concentration range, MI showed a strong negative concentration–response relationship (r = −0.84) (Figure 1a), and the 500 and 1000 µg/mL concentrations fell outside the OECD 487 acceptable cytotoxicity window.

In the micronucleus test prepared with IPF-BH exposure (48 h effective treatment, in line with the cytokinesis-block protocol of OECD 487), micronucleus frequencies were determined by examining 2000 binucleate cells per concentration group (500 binucleate cells per donor across four donors). NDI values were calculated in parallel and remained interpretable across all retained concentrations (data in line with the MN scoring shown in Table 2). As summarized in Table 2, an increase in the micronucleus frequency was observed with increasing concentration.

**Figure 2:**
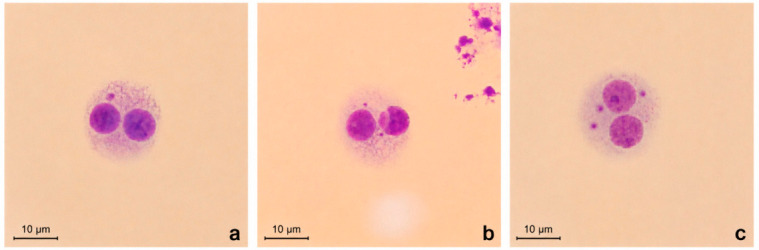
Binucleated cells with 1 micronucleus ((**a**), 62.5 μg/mL), 2 micronuclei ((**b**), 500 μg/mL), and 3 micronuclei ((**c**), 1000 μg/mL) observed at application concentrations.

The MN frequency increase was statistically significant at all IPF-BH concentrations except 62.5 µg/mL. Cells with one, two, three and more micronuclei were detected among binucleate cells (Figure 2), reflecting varying degrees of chromosomal damage or mitotic dysfunction. No apoptotic cells were observed among the micronucleated cells. The MN frequencies were strongly correlated with concentration (r = 0.96) (Figure 1b). However, the two highest concentrations (500 and 1000 µg/mL) exceeded the OECD 487 cytotoxicity threshold (Table 1, relative MI < 65%), and the corresponding MN increases at these concentrations should therefore be interpreted as cytotoxicity-associated rather than as evidence of direct clastogenic/aneugenic activity; the MN increases at 125 and 250 µg/mL, although obtained at concentrations close to or just below the OECD threshold, are more consistent with a cytogenetic effect that nevertheless cannot be unambiguously attributed to direct DNA damage without additional mechanistic data.

DNA damage in human lymphocytes following *in vitro* exposure to IPF-BH was further quantified using the comet assay, and the results are summarized in Table 3.

**Figure 3:**
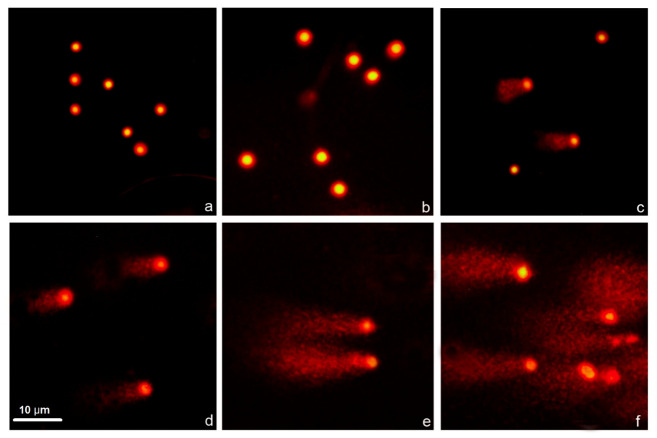
Comets with no damage, moderate damage, and severe damage belonging to negative controls (**a**,**b**), application concentrations (**c**,**d**), and positive controls (**e**,**f**).

Significant alterations were observed in comet assay parameters, including tail length, tail intensity, and tail moment, compared to the status of the negative control, indicating increased DNA strand breaks and overall genomic damage in treated cells (Figure 3). Concentration–response analysis revealed different levels of correlation between comet assay parameters and increasing concentrations. Tail intensity (r = 0.32) and tail length (r = 0.35) showed weak correlations, indicating no strict concentration-dependent relationship. In contrast, the tail moment exhibited a stronger concentration-dependent correlation (r = 0.67) (Figure 1c,d,e).

The concentration–response pattern of the comet assay parameters was not strictly monotonic. In particular, the highest mean tail length (8.13 ± 0.81 µm) was observed at 250 µg/mL of IPF-BH, while the corresponding values at 500 µg/mL (4.84 ± 0.23 µm) and 1000 µg/mL (6.03 ± 0.56 µm) were lower. A similar, although less pronounced, non-monotonicity was observed for tail intensity. This pattern is consistent with the strongly cytotoxic nature of the highest concentrations (relative MI < 65%, Table 1) and may reflect selective loss of severely damaged or apoptotic cells from the scoring population, either by detachment from slides during lysis/electrophoresis or by exclusion of “hedgehog”/“ghost” comets that are not considered representative of repairable DNA damage [31,32]. Such selective loss can disproportionately reduce mean tail length while permitting mean tail intensity and tail moment to remain relatively higher. Severe cytotoxicity-associated chromatin condensation and inter-strand cross-linking can also shorten the apparent comet tail without reducing total DNA damage. The per-concentration scoring depth (100 cells per donor; 400 cells per group) is intrinsically less robust than are larger scoring strategies against such outlier-loss effects. Tail moment, which integrates both tail length and tail intensity, is therefore regarded as the most reliable index of the concentration–response relationship in the present dataset, while the comet response as a whole should be interpreted as a cytotoxicity-associated genotoxic-like signal rather than as direct evidence of clastogenic/strand-break-inducing genotoxicity.

## 4. Discussion

This study investigated the *in vitro* cytotoxic and cytogenetic responses of an ipfencarbazone-based herbicide (IPF-BH) in human peripheral lymphocytes using mitotic index (MI), micronucleus (MN), and comet assay endpoints. Overall, the findings demonstrate a concentration-dependent suppression of mitotic activity, accompanied by increased MN formation and elevated DNA damage parameters. In view of the strong cytotoxicity observed at the upper concentrations (relative MI of 50–62% at 500–1000 µg/mL of IPF-BH, i.e., exceeding the OECD 487 cytotoxicity threshold of ~55 ± 5%) and in view of the fact that the test substance was a commercial formulation rather than analytical-grade ipfencarbazone (see Section 4.1 below), the cytogenetic responses described here are best interpreted as cytotoxicity-associated genotoxic-like effects rather than as direct genotoxicity.

The significant reduction in MI across all tested concentrations, culminating in a complete inhibition of cell division at the highest dose, confirms that IPF-BH exerts a marked cytotoxic effect on human lymphocytes. Ipfencarbazone, the active ingredient of IPF-BH, has been reported to inhibit the biosynthesis of very-long-chain fatty acids (VLCFAs) in plants [11]. Given the essential role of VLCFAs in maintaining membrane integrity and cellular homeostasis, disruption of this pathway may plausibly contribute to membrane destabilization and impaired cell division in mammalian cells; this remains hypothetical in the present study, since no direct membrane-integrity measurements (e.g., LDH leakage) were performed. Consistent with this, other herbicides within the triazolinone class have been shown to induce cytotoxic effects in various cellular systems [33,34].

The observed increase in MN frequency, particularly at higher concentrations, may indicate the involvement of clastogenic and/or aneugenic mechanisms. Micronuclei arise from chromosome fragments or whole chromosomes that fail to be incorporated into daughter nuclei during mitosis. However, as emphasized in OECD Guideline 487, increases in MN frequency under conditions of excessive cytotoxicity may represent secondary effects rather than direct genotoxic events [35]. In the present study, the MN response at 500 and 1000 µg/mL—where the relative MI values fell below the OECD 487 cytotoxicity threshold—should therefore not be interpreted as evidence of direct genotoxicity. The MN increases at the intermediate concentrations (125 and 250 µg/mL), although closer to the acceptable cytotoxicity window, also occurred in the context of a clear concomitant MI reduction and are most appropriately described as cytotoxicity-associated genotoxic-like effects. Similar increases in MN formation have been reported for pesticides such as sulfentrazone and imidacloprid in *in vitro* systems [8,36].

Comet assay results revealed significant increases in DNA damage parameters across all tested concentrations. In particular, elevations in tail intensity and tail moment are commonly associated with DNA strand breaks [31]. The more consistent concentration-dependent relationship observed for tail moment may indicate its relative sensitivity in reflecting the extent of DNA damage and is consistent with the non-monotonic behavior of tail length and tail intensity discussed above. Nevertheless, DNA damage detected by the comet assay may arise not only from direct genotoxic interactions but also from indirect mechanisms associated with cytotoxicity, including apoptosis and oxidative stress [32]. Indeed, several herbicides have been shown to induce reactive oxygen species (ROS) production, leading to oxidative DNA damage [37,38]. In the present study, however, ROS generation was not measured directly, nor were other oxidative stress markers (lipid peroxidation, antioxidant enzyme activity, reduced/total glutathione, mitochondrial transmembrane potential, or membrane integrity). The contribution of oxidative stress to the observed comet response therefore remains hypothetical and is presented here only as a candidate mechanism consistent with the evidence in the wider literature on related triazolinone herbicides.

From a mechanistic perspective, it has been hypothesized that inhibition of VLCFA biosynthesis by ipfencarbazone could provide a plausible basis for indirect cytogenetic outcomes through disruption of membrane integrity, mitochondrial dysfunction, and oxidative stress pathways [11,33]. We emphasize, however, that in the present study, none of these mechanistic endpoints—intracellular ROS, mitochondrial transmembrane potential, lipid peroxidation, glutathione status, or membrane integrity—was measured directly. The above mechanisms are therefore best regarded as candidate pathways consistent with the literature on related triazolinone herbicides rather than as mechanisms demonstrated for IPF-BH. In addition, in silico predictions have suggested potential mutagenic and toxicological liabilities for ipfencarbazone, which appear to be partially consistent with the present *in vitro* findings. However, carcinogenicity-related outcomes remain uncertain and were not directly addressed in this study. While the experimental data may lend some support to computational predictions, further investigation is required to clarify the carcinogenic potential of this compound [26]. Taken together, the present data describe cytotoxicity and cytotoxicity-associated genotoxic-like effects of IPF-BH in human peripheral lymphocytes but do not establish direct genotoxicity of either the formulation or its active ingredient. Within the indicated *in vitro* model, however, the concentration-dependent increases in MN frequency and in alkaline-comet parameters observed at concentrations falling within the OECD 487 acceptable cytotoxicity window (62.5–250 µg/mL of IPF-BH; relative MI ≥ ~66%, Table 1) do constitute a genotoxic response of IPF-BH in this *in vitro* model, while the corresponding increases at the strongly cytotoxic concentrations (500 and 1000 µg/mL; relative MI < 65%) are most appropriately interpreted as cytotoxicity-associated genotoxic-like effects, in line with OECD 487 guidance.

A separate, and scientifically central, question concerns the reported discrepancy between negative or weakly positive genotoxicity findings for ipfencarbazone in standard *in vivo* rodent studies [10] and the positive cytogenetic responses observed in cultured cells, including those reported in the present manuscript. Several mechanistic factors plausibly contribute to this apparent *in vitro*/*in vivo* discordance. First, species differences in the xenobiotic-metabolizing enzyme repertoire (phase I cytochrome P450 isoforms and phase II conjugating enzymes) between rodents and humans, and between primary cultured cells and the intact liver, can substantially alter the balance between the bioactivation of ipfencarbazone and its detoxification. Second, the present *in vitro* design—using primary human peripheral lymphocytes without exogenous metabolic activation (no S9 supplementation)—predominantly reflects the cytotoxic and cytogenetic behavior of the parent IPF-BH formulation (and its co-formulants) in the absence of meaningful CYP-mediated biotransformation; this is itself one of the experimental conditions historically associated with positive *in vitro*/negative *in vivo* discordance for several pesticide-related compounds. Third, intact *in vivo* systems benefit from systemic detoxification (hepatic phase II conjugation, biliary and urinary clearance) and from compartmentalization, both of which can reduce target-tissue exposure to the parent compound and to its bioactivated metabolites in a manner that is not reproducible in a static *in vitro* lymphocyte culture. Fourth, primary human peripheral lymphocytes possess only limited intrinsic CYP-mediated biotransformation capacity, so that a +S9/−S9 comparison is required to discriminate parent-compound effects from metabolite-mediated effects. The present study cannot resolve this *in vitro*/*in vivo* discordance on its own, and a coordinated bridging strategy combining parallel +S9/−S9 *in vitro* experiments (with rat liver S9 and, where feasible, with human-relevant metabolic activation systems such as cryopreserved hepatocytes or HepaRG-conditioned media) with OECD TG 474/475/489-compliant *in vivo* rodent follow-up studies is proposed as the appropriate path forward (see Section 4.4).

The cytogenetic endpoints employed in the present study—the cytokinesis-block MN assay and the alkaline comet assay—were selected as complementary methods covering chromosome-level (clastogenic/aneugenic) damage and primary DNA strand breaks, respectively, in accordance with the OECD genotoxicity testing battery (OECD TG 487 and OECD TG 489). The classical chromosomal aberration (CA) analysis (OECD TG 473), which directly visualizes structural chromosome damage at metaphase, was not performed in this study, primarily due to the pronounced reduction in the number of mitotic figures at the highest concentrations; the substantial donor blood volume required to combine MI, MN, comet, and CA endpoints; and the partially overlapping mechanistic information already provided by the MN assay. The absence of CA analysis represents a limitation of the present work, and future studies on IPF-BH and on analytical-grade ipfencarbazone should include CA scoring of chromosome- and chromatid-type aberrations (OECD TG 473) in order to provide a more comprehensive structural cytogenetic profile and to better discriminate between clastogenic and aneugenic modes of action.

A further methodological consideration concerns the cytostasis parameter used in the cytokinesis-block MN assay. In the present study, cytostasis and cell-cycle progression were evaluated using the nuclear division index (NDI), a parameter that has been widely applied in cytokinesis-block MN assays of human peripheral lymphocytes and that is well established in the cytogenetic literature, including in studies based on the HUMN (Human Micronucleus) Project protocols. We acknowledge, however, that the current version of OECD Test Guideline 487 explicitly recommends the cytokinesis-block proliferation index (CBPI) as the preferred parameter for quantifying cytotoxicity/cytostasis in this assay system, given its closer correspondence to relative population doubling and its direct linkage to the OECD cytotoxicity threshold of 55 ± 5%. Although NDI and CBPI provide overlapping information on cell-cycle progression, the absence of a formal CBPI calculation in the present study is a methodological limitation, and CBPI determination should be incorporated in future studies aiming for full OECD 487 compliance.

In addition, the selection of the highest test concentration deserves explicit consideration. The initial concentration series in the present study was guided by the reported acute LD50 value of ipfencarbazone in rats (2000 mg/kg), and the highest applicable concentration in the *in vitro* system was empirically rescaled to 1000 µg/mL after preliminary observations showed complete inhibition of cell division at 2000 µg/mL. Although the retained concentration range (62.5–1000 µg/mL) allowed a clear concentration–response analysis, it is not fully compliant with the cytotoxicity-based concentration selection criteria of OECD 487, which recommends selecting the highest concentration so that cytotoxicity does not exceed approximately 55 ± 5%. As described in the Section 3, the relative MI values at 500 and 1000 µg/mL fall below this threshold, and the cytogenetic responses observed at these concentrations are therefore most appropriately interpreted as cytotoxicity-driven rather than as evidence of direct genotoxicity.

### 4.1. Formulation Considerations

An important interpretative caveat is that the test material in the present study was a commercial ipfencarbazone-based herbicide formulation (IPF-BH; Hokuto) rather than analytical-grade ipfencarbazone. Commercial herbicide formulations typically contain, in addition to the declared active ingredient, a range of co-formulants such as surfactants, emulsifiers, solvents, and stabilizers, the identities and concentrations of which are generally considered confidential business information and were not disclosed by the manufacturer of Hokuto. A growing body of literature has shown that the cytotoxic, and in some cases, the genotoxic activity of pesticide formulations can substantially exceed that of the corresponding pure active ingredients, an effect frequently attributed to surfactants and adjuvants that increase membrane permeability and intracellular uptake. Within the present design, it is therefore not possible to disentangle the cytotoxic and cytogenetic contributions of ipfencarbazone itself from those of the IPF-BH co-formulants, and the results should be interpreted as describing the toxicological behavior of the formulation as a whole rather than of the active ingredient in isolation. This limitation is further addressed in Section 4.2 and is reflected in the recommendations for future work.

### 4.2. Environmental and Occupational Relevance of the Tested Concentrations

The tested IPF-BH concentration range (62.5–1000 µg/mL) was selected as an *in vitro* hazard-identification range, anchored to the acute oral LD50 of ipfencarbazone in rats (2000 mg/kg) [10] and constrained by the OECD 487 cytotoxicity criteria, as discussed above. These concentrations clearly exceed available environmental dietary exposure levels for ipfencarbazone, which include residue concentrations of approximately 0.01 mg/kg in fishery products [16] and up to 14.71 mg/kg in rice after application at 312.50 g a.i./ha [30], together with an established acceptable daily intake (ADI) of 0.00099 mg/kg body weight per day [14,15]. They should therefore not be interpreted as representative of chronic dietary intake. They may, however, be more relevant to acute, high-level occupational exposure scenarios—for example, dermal or inhalation contact during mixing, loading, and application of IPF-BH in paddy rice cultivation—where localized peripheral lymphocyte exposure to elevated concentrations cannot be excluded, particularly in the absence of adequate personal protective equipment. The present data should therefore be regarded as exploratory hazard-identification information for the formulation, intended to support, but not to replace, biomonitoring and dose-relevant follow-up studies in occupationally exposed populations.

### 4.3. Limitations

Several limitations of this study should be considered. First, the absence of a metabolic activation system (S9 fraction) limits the assessment of potential bioactivation and metabolite-mediated effects [39]. Human peripheral lymphocytes possess only limited intrinsic xenobiotic-metabolizing enzyme activity (e.g., modest CYP-mediated capacity), so that experiments conducted in the absence of an exogenous metabolic activation system reflect the toxicological behavior of the parent active ingredient and the formulation co-formulants as applied, but do not address the potential cytotoxicity or cytogenetic effects of metabolite-mediated species (e.g., CYP-generated metabolites). Within the OECD 487 framework, parallel testing with and without an exogenous metabolic activation system is therefore typically required for regulatory hazard identification in order to capture both parent-compound and metabolite-mediated effects, and the present results should accordingly be regarded as informative for the unactivated parent formulation only. Second, the study was not conducted under good laboratory practice (GLP) conditions, which may limit the regulatory applicability of the findings [40]. Third, the marked cytotoxicity observed at higher concentrations—with the relative MI falling below the OECD 487 acceptable threshold at 500 and 1000 µg/mL—strongly influenced the interpretation of the cytogenetic endpoints; in line with OECD 487 recommendations, the MN and comet responses observed at these concentrations cannot be unambiguously attributed to direct genotoxicity. Fourth, the MN and comet assays were each carried out at a single, assay-specific exposure duration (effective 48 h for MN and 1 h for the alkaline comet assay), and the time-dependence of IPF-BH–induced cytogenetic damage was not systematically evaluated. Fifth, cytostasis was assessed using NDI rather than the CBPI parameter recommended by OECD 487, and the classical chromosomal aberration analysis (OECD TG 473) was not performed, as detailed above. Sixth, although the total per-concentration scoring strategy (4000 cells for MI, 2000 binucleate cells for MN, 400 cells for the comet assay) is consistent with cumulative-scoring practice in pooled-donor cytogenetic studies, the per-donor cell numbers used here (500 binucleate cells/donor for MN and 1000 cells/donor for NDI) are below the per-donor thresholds recommended in OECD TG 487 (2000 binucleate cells per donor for MN and 500 cells per donor for CBPI). Seventh, peripheral blood was obtained from only four healthy adult donors (two male and two female), and the data were pooled across donors per concentration. This is acknowledged as a small sample size that limits the statistical robustness and external generalizability of the findings; inter-individual variability in baseline and induced DNA damage responses in human peripheral lymphocytes—reflecting differences in DNA repair capacity, antioxidant status, lifestyle factors, and polymorphisms in xenobiotic-metabolizing enzymes—may not be adequately captured by such a small cohort, and the results should therefore be regarded as exploratory rather than as a definitive characterization of population-level risk. A formal sex-stratified statistical comparison was likewise not conducted, as the limited per-sex donor number (*n* = 2 males, *n* = 2 females) is not sufficient to support robust between-sex statistical inference. Eighth, no direct mechanistic measurements—including intracellular ROS generation, mitochondrial transmembrane potential, lipid peroxidation, antioxidant enzyme activity, reduced/total glutathione, or membrane integrity assays—were performed; consequently, the mechanistic discussion in this study is framed as hypothetical, drawing on the wider literature on related triazolinone herbicides rather than on direct experimental evidence in the present model, and the present data alone do not support definitive conclusions regarding the mechanism of action of IPF-BH. Finally, as detailed in Section 4.1, the use of a commercial IPF-BH formulation rather than analytical-grade ipfencarbazone means that the present findings cannot be unambiguously attributed to the active ingredient alone, and a contribution of undisclosed co-formulants cannot be excluded. In addition, cytotoxicity in the present study was monitored via the mitotic index (MI), supplemented with the nuclear division index (NDI) for cytostasis, the relative MI as the OECD 487 cytotoxicity-window indicator, and trypan-blue exclusion viability at the pre-exposure lymphocyte isolation step (>99% viability, Section 2.5). We acknowledge that MI alone is not a complete cytotoxicity indicator, since it primarily reflects proliferative suppression rather than post-treatment cell viability or membrane integrity; a complete OECD 487-aligned cytotoxicity characterization would additionally include at least one parallel post-treatment viability or membrane-integrity assay (e.g., MTT, resazurin, propidium-iodide/7-AAD exclusion, or LDH leakage), and ideally, CBPI/relative population doubling. The conjoint use of MI + NDI + relative MI + pre-exposure trypan-blue viability in the present study should therefore be regarded as a partial—not complete—cytotoxicity characterization.

### 4.4. Future Perspectives

Future studies on the cytotoxic and cytogenetic potential of ipfencarbazone and IPF-BH should ideally (i) be performed in parallel with and without an exogenous metabolic activation system (rat liver S9 fraction) and, where feasible, with human-relevant metabolic activation systems such as cryopreserved hepatocytes or HepaRG-conditioned media, in order to properly assess the metabolic-activation profile of the compound and capture metabolite-mediated cytotoxic and cytogenetic effects, (ii) employ a wider range of exposure durations—in particular, extended treatment intervals such as 24 h and 48 h in the alkaline comet assay and longer treatment windows in the MN assay—to characterize the time-dependence of cytogenetic damage, (iii) incorporate OECD TG 473-compliant chromosomal aberration analysis to complete the structural cytogenetic profile, (iv) apply the CBPI parameter as recommended by OECD TG 487 for cytostasis evaluation, (v) increase the per-donor scoring depth to meet OECD per-donor thresholds for MN and CBPI, (vi) recruit a substantially larger and sex-balanced cohort of donors to enable robust per-donor and sex-stratified analyses and to better capture inter-individual variability in lymphocyte DNA damage responses, (vii) systematically incorporate direct mechanistic endpoints in the same cell system—including intracellular ROS measurement (e.g., DCFH-DA), mitochondrial transmembrane potential (e.g., JC-1 or TMRE), lipid peroxidation (e.g., TBARS/MDA), antioxidant enzyme activity (e.g., superoxide dismutase, catalase, glutathione peroxidase), reduced/total glutathione (GSH/GSSG), and membrane integrity assays (e.g., LDH leakage)—in order to mechanistically anchor the observed cytogenetic effects, (viii) test analytical-grade ipfencarbazone, the full IPF-BH formulation, and a “formulation-blank” (co-formulants without the active ingredient) in parallel so that the cytotoxic and cytogenetic contributions of the active ingredient and the co-formulants can be disentangled, (ix) restrict the genotoxicity-evaluation concentration range to within the OECD 487 cytotoxicity window to enable a clearer separation of direct genotoxic effects from cytotoxicity-driven artefacts, and (x) extend the dose range to concentrations more representative of realistic environmental dietary exposure, together with biomonitoring data from occupationally exposed populations. Complementary *in vivo* follow-up studies and mechanistic investigations focused on oxidative stress, mitochondrial function, and VLCFA-related membrane integrity would further refine the toxicological profile of this compound. Finally, we note that the primary literature directly addressing the toxicology of ipfencarbazone in mammalian and human cells remains very limited, with most available information confined to its agronomic development [10,11,12,13], environmental fate and residues [16,17,18,30], regulatory safety data sheets [14,15], and *in silico* predictions [26]; this limited evidence base is itself a strong motivation for further targeted mammalian and human-cell studies of this active ingredient.

In addition, future IPF-BH studies in primary human peripheral lymphocytes and in other cellular models should routinely complement the mitotic index with at least one parallel post-treatment cytotoxicity/viability readout—for example, MTT or resazurin metabolic-viability assays, post-treatment trypan-blue exclusion or propidium-iodide/7-AAD live/dead staining, LDH-leakage membrane-integrity assays, and the OECD 487-recommended cytokinesis-block proliferation index (CBPI) or relative population doubling (RPD)—in order to provide a complete cytotoxicity characterization and to enable a cleaner separation of direct genotoxic effects from cytotoxicity-driven secondary signals.

### 4.5. Conclusions

In conclusion, IPF-BH induced marked cytotoxic effects in human peripheral lymphocytes under *in vitro* conditions and was associated with increased micronucleus formation and DNA damage parameters. Within the indicated *in vitro* model, the concentration-dependent increases in MN frequency and in alkaline-comet parameters observed at concentrations within the OECD 487 acceptable cytotoxicity window (62.5–250 µg/mL of IPF-BH) constitute a genotoxic response of IPF-BH in this *in vitro* model, while the corresponding responses at the strongly cytotoxic concentrations (500 and 1000 µg/mL; relative MI below the OECD 487 acceptable threshold), in the absence of direct mechanistic measurements and given the use of a commercial formulation rather than analytical-grade ipfencarbazone, are most appropriately described as cytotoxicity-associated genotoxic-like effects. Because the present design was instituted without an exogenous bioactivating system, extrapolation of the *in vitro* genotoxic response to the *in vivo* situation requires complementary +S9 *in vitro* experiments and OECD TG 474/475/489-compliant *in vivo* rodent follow-up studies. Further studies incorporating metabolic activation systems and *in vivo* models, together with chromosomal aberration analysis, CBPI-based cytostasis evaluation, extended exposure durations, OECD-compliant cytotoxicity-windowed concentration ranges, direct mechanistic endpoints (ROS, mitochondrial function, lipid peroxidation, antioxidant enzyme activity, glutathione status, membrane integrity), parallel post-treatment viability/membrane-integrity assays (e.g., MTT, trypan-blue exclusion, LDH leakage), parallel testing of analytical-grade ipfencarbazone versus the formulation, and a larger sex-balanced donor cohort, are warranted to better characterize the toxicological profile of IPF-BH and of ipfencarbazone as an active ingredient.

## Figures and Tables

**Table 1:** Mitotic index frequency data according to concentrations of IPF-BH after 24 h of exposure.

Test Substance	Treatment Period (h)	Concentration (µg/mL)	Counted Cells	MI Frequency ± SE (%)	Relative MI (%)
Negative Control	24	0	4000	5.85 ± 0.43	100.0
IPF-BH	24	125	4000	4.60 ± 0.33 ***	78.6
		250	4000	3.90 ± 0.27 ***	66.7
		500	4000	3.65 ± 0.20 ***	62.4
		1000	4000	2.95 ± 0.15 ***	50.4
		2000	0 ^a^	0 ^a^	0.0
Positive Control (MMC)	24	0.2	4000	2.15 ± 0.24 ***	36.8

Statistical significance versus negative control (one-way ANOVA followed by Dunnett’s post hoc test): *** *p* < 0.001. ^a^ No dividing cells were observed at 2000 µg/mL due to high cytotoxicity; this concentration was therefore excluded from quantitative MI comparison and from subsequent cytogenetic assays. Relative MI was calculated as (MI of treated group/MI of negative control) × 100%; values below ~45% correspond to cytotoxicity above the OECD 487 threshold of approximately 55 ± 5%. SE, standard error. IPF-BH, ipfencarbazone-based herbicide.

**Table 2:** Micronucleus frequency data according to concentrations of IPF-BH after 48 h of effective exposure.

Test Substance	Treatment Period (h)	Concentration (μg/mL)	BN Cells Scored	MN Count	MN (%) ± SE
Negative Control	48	0	2000	13	0.6 ± 0.17
IPF-BH	48	62.5	2000	14	0.7 ± 0.19
		125	2000	28	1.4 ± 0.26 *
		250	2000	34	2.0 ± 0.31 ***
		500	2000	46	2.9 ± 0.37 ***
		1000	2000	59	3.8 ± 0.42 ***
Positive Control (MMC)	48	0.2	2000	111	6.4 ± 0.45 ***

Statistical significance versus negative control (one-way ANOVA, followed by Dunnett’s post hoc test): * *p* < 0.05 and *** *p* < 0.001. BN, binucleated cells; MN, micronuclei; SE, standard error. IPF-BH, ipfencarbazone-based herbicide.

**Table 3:** Assessment of DNA damage using the alkaline comet assay after *in vitro* exposure of human lymphocytes to IPF-BH.

Test Substance	Concentration (µg/mL)	Tail Length (µm)	Tail Intensity (%)	Tail Moment
Negative Control	0	3.34 ± 0.15	3.90 ± 0.11	0.18 ± 0.22
IPF-BH	62.5	4.14 ± 0.25 *	9.77 ± 0.72 ***	2.54 ± 0.36 ***
	125	5.07 ± 0.52 *	6.19 ± 0.59 ***	1.81 ± 0.25 ***
	250	8.13 ± 0.81 ***	8.49 ± 0.74 ***	1.93 ± 0.27 ***
	500	4.84 ± 0.23 ***	8.99 ± 0.34 ***	2.89 ± 0.29 ***
	1000	6.03 ± 0.56 ***	7.89 ± 0.36 ***	2.99 ± 0.29 ***
Positive Control (H_2_O_2_)	3.4	10.55 ± 1.32 ***	35.15 ± 0.85 ***	4.16 ± 0.69 ***

Values represent the analysis of 400 cells per concentration group (100 cells per donor across four donors). Tail length is expressed in µm, tail intensity (%) as the percentage of DNA in the tail, and tail moment as the product of tail length and tail intensity. Data are presented as mean ± SE. Statistical significance versus negative control (one-way ANOVA, followed by Dunnett’s post hoc test): * *p* < 0.05 and *** *p* < 0.001. IPF-BH, ipfencarbazone-based herbicide.

## Data Availability

The original contributions presented in this study are included in the article. Further inquiries can be directed to the corresponding author.

## Abbreviations

The following abbreviations are used in this manuscript:

PPO	Protoporphyrinogen Oxidase
VLCFA	Very-Long-Chain Fatty Acid
MMC	Mitomycin-C
EtBr	Ethidium Bromide
MI	Mitotic Index
MN	Micronuclei
NDI	Nuclear Division Index
CBPI	Cytokinesis-Block Proliferation Index
CA	Chromosomal Aberration
EC50	Effective Concentration 50
GSI	Gonadosomatic Index
OECD	Organization for Economic Co-Operation and Development
BN	Binucleate
SE	Standard Error
IPF-BH	Ipfencarbazone-Based Herbicide (commercial formulation Hokuto, 250 g/L ipfencarbazone)
ROS	Reactive Oxygen Species
ADI	Acceptable Daily Intake
ANOVA	Analysis of Variance
DMSO	Dimethyl Sulfoxide
EDTA	Ethylenediaminetetraacetic Acid
